# ASSOCIATION BETWEEN GAIT SPEED MEASURED USING A WEARABLE DEVICE AND SARCOPENIA

**DOI:** 10.1093/geroni/igz038.3242

**Published:** 2019-11-08

**Authors:** Min-gu Kang, Kwang-il Kim, Joon Koo Kang, Seong-Ji Kang, Hye-Kang Roh, Hwa-Young Jung

**Affiliations:** 1 Department of Internal Medicine, Chonnam National University Bitgoeul Hospital, Gwangju, Korea, Republic of; 2 Seoul National Bundang Hospital, Bundang-gu, Seongnam-si, Korea, Republic of; 3 Graduate School of Health Science and Management, Yonsei University, Seoul, Korea, Republic of; 4 WELT Corp., Ltd, Seoul, Korea, Republic of

## Abstract

As slow gait speed is a major feature of frailty and a diagnostic criterion of sarcopenia, gait speed measurement is widely used. Nowadays, with development of wearable devices, it is possible to measure daily-life gait speed without additional effort just by wearing the device. It is meaningful to measure daily-life gait speed and to analyze the association between the speed and sarcopenia. Participants were men over 50 years of age who visited the university hospital. Daily-life gait speed was checked using a smart belt (WELT) for 4 weeks. Afterwards, a survey about past medical history, usual gait speed measurement, handgrip strength measurement, and dual energy X-ray absorptiometry were performed. A total of 217,548 daily-life gait speed measurement data were analyzed for 106 participants. The mean daily-life gait speed was 1.23 ± 0.26 m/s. The mean age was 71.1 ± 7.6, and daily-life gait speed was significantly slower as people get older. (P<0.001) Additionally, weekday gait speed (1.23 ± 0.26 m/s) was significantly faster than weekend gait speed (1.22 ± 0.26 m/s). (P<0.001) Participants with sarcopenia (1.15 ± 0.25 m/s) had significantly slower mean daily-life gait speed than normal subjects (1.23 ± 0.26 m/s). (P<0.001) In analyzing factors related to gait speed, age and skeletal muscle mass of lower limbs were significantly associated with mean daily-life gait speed. Additional information about the gait speed can be obtained by measuring daily-life gait speed, and the daily-life gait speed has a significant association with the skeletal muscle mass of lower limbs.

